# Supplementary material for: Multiscale interactome analysis coupled with off-target drug predictions reveals drug repurposing candidates for human coronavirus disease
Source: Sci Rep. 2021 Dec 2;11:23315. doi: 10.1038/s41598-021-02432-7 (PMC8640055; doi:10.1038/s41598-021-02432-7)
Supplement: Supplementary file 1 — Supplementary Information. [file 41598_2021_2432_MOESM1_ESM.pdf]

Sugiyama MG, Cui H, Redka DS, et al. Supplementary Information

This Supplementary Information contains

Table S1
Table S2
Table S3
Table S4
Figure S1
Figure S2
Figure S3
Figure S4

Table S1. Cell culture reagents for propagation of human coronaviruses

Name	Component	Company	Catalogue #	Final Amount
**Growth Media**	Minimum Essential Media Eagle	Millipore Sigma	M4655	N/A
	Fetal Bovine Serum, Heat-inactivated	Thermo Fisher	10082147	10%
	Penicillin-Streptomycin	Thermo Fisher	15070-063	1X
**Infection Media**	Minimum Essential Media Eagle	Millipore Sigma	M4655	N/A
	Fetal Bovine Serum, Heat-inactivated	Thermo Fisher	10082147	2%
	Penicillin-Streptomycin	Thermo Fisher	15070-063	1X
**Plaque Media**	MEM (Temin's modification) (2X), no phenol red	Thermo Fisher	11935046	1X
	Fetal Bovine Serum, Heat-inactivated	Thermo Fisher	10082147	1%
	Penicillin-Streptomycin	Thermo Fisher	15070-063	1X
	0.6% Agarose	Thermo Fisher	16500-500	0.3%
**Transfection Master Mix**	Opti-MEM- I Reduced-Serum Medium	Thermo Fisher	31985070	N/A
	Lipofectamine RNAiMAX	Thermo Fisher	13778030	6.3 µL per well
	siRNA (control or target)	N/A	N/A	50nM

Table S2. Oligonucleotide sequences for siRNA transfection or qPCR

Target	Forward (5' to 3')	Reverse (5' to 3')	Application
NL63 N	GATAACCAGTCGAAGTCACCTAGTTC	ATTAGGAATCAATTCAGCAAGCTGTG	qPCR
GAPDH	GTCTCCTCTGACTTCAACAGCG	ACCACCCTGTTGCTGTAGCCAA	qPCR
IRAK1	CGAAGAAAGUGAUGAAUUUUU	AAAUUCAUCACUUUCUUCGUU	siRNA
IRAK4	CUUUGAUGAACGACCCAUUUU	AAUGGGUCGUUCAUCAAAGUU	siRNA
Control	CGUACUGCUUGCGAUACGGUU	CCGUAUCGCAAGCAGUACGUU	siRNA

Table S3. RT-qPCR protocol

Step	Temperature	Time
1. Reverse transcription	55°C	10 minutes
2. Hot start	95°C	1 minute
3. Denaturation	55°C	10 seconds
4. Extension (with plate read)	62°C	30 seconds
5. Repeat 3-5 (45x)	N/A	N/A
6. Melt curve	60-95°C, 0.5°C increments	5 seconds

Table S4. Additional information for compounds used in this study

Number	Compound	Supplier	Cat. No.	Final Concentration
1	Bucladesine	MedChemExpress	HY-B0764	10 µM
2	Cinnarizine	MedChemExpress	HY-B1090	1 µM
3	Doxycycline	MedChemExpress	HY-N0565	10 µM
4	Eflornithine	MedChemExpress	HY-B0744	10 µM
5	Flucytosine	MedChemExpress	HY-B0139	10 µM
6	Opicapone	MedChemExpress	HY-14896	1 µM
7	Otilonium Bromide	MedChemExpress	HY-B0499A	1 µM
8	Cefotiam	MedChemExpress	HY-B0734A	1 µM
9	Dapagliflozin	MedChemExpress	HY-10450	1 µM
10	Fosamprenavir	MedChemExpress	HY-78726	10 µM
11	Gentamicin	MedChemExpress	HY-A0276	10 µM
12	Glipizide	MedChemExpress	HY-B0254	10 µM
13	Palbociclib	MedChemExpress	HY-50767	1 µM
14	Saquinavir	MedChemExpress	HY-17007	1 µM
15	Streptozotocin	MedChemExpress	HY-13753	10 µM
16	Sugammadex	MedChemExpress	HY-B0079	10 µM
17	Tofacitinib	MedChemExpress	HY-40354	10 µM
18	Capmatinib	MedChemExpress	HY-13404	10 µM
19	Anidulafungin	MedChemExpress	HY-13553	1 µM
20	Glibenclamide	MedChemExpress	HY-15206	10 µM
21	Nelarabine	MedChemExpress	HY-13701	1 µM
22	Remdesivir	MedChemExpress	HY-104077	4 µM
23	Degarelix	MedChemExpress	HY-16168A	1 µM
24	JH-I-25	Millipore-Sigma	SML2609	10 µM
25	SP600125	Millipore-Sigma	S5567	10 µM
26	SB202190	abcam	ab120638	10 µM
27	PD98059	abcam	ab120234	10 µM

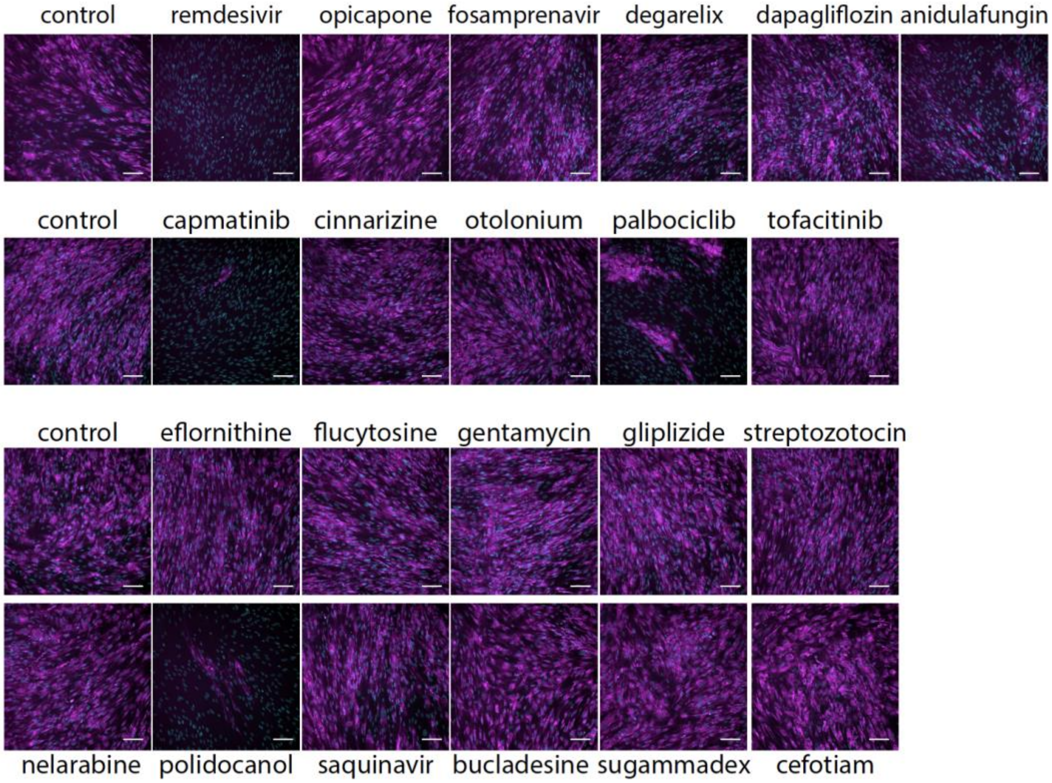

Figure S1. Representative microscopy images for data shown in Figure 2. Representative images from MRC-5 cells treated with drugs as indicated (see **Table S4**) and infected with 229E for 2 days. Images depict S protein expression (magenta) or DAPI (cyan) Scale, 100 µm.

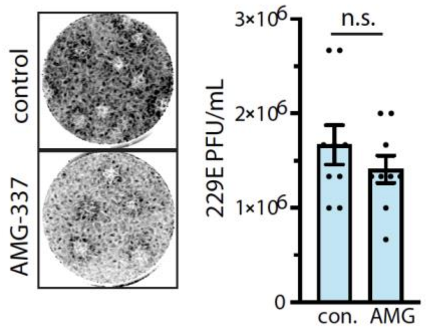

Figure S2. Inhibition of MET by AMG337 does not impact 299E infection assessed by PFU assay. (left) Representative images of plaques in MRC-5 cells treated with DMSO (vehicle) control or 10 µM AMG-337 and infected with 299E and (right) quantification of 299E viral titer shown as mean PFU ± SE (n = 3 with 3 technical replicates per experiment).

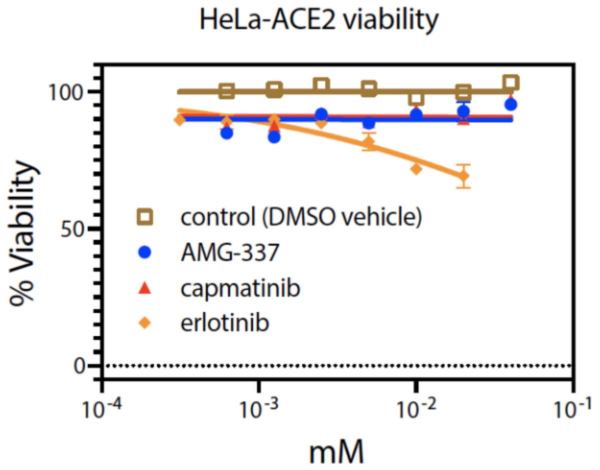

Figure S3. Capmatinib does not affect cell viability. HeLa-ACE2 cells were treated with inhibitors as shown (10 µM each) in parallel with the PsV assay shown in Figure 4D. Shown are representative cell viability measurements from n=3 independent experiments. Neither capmatinib nor AMG-337 impact cell viability. In contrast, the EGF Receptor inhibitor erlotinib resulted in a dose-dependent impairment of cell viability.

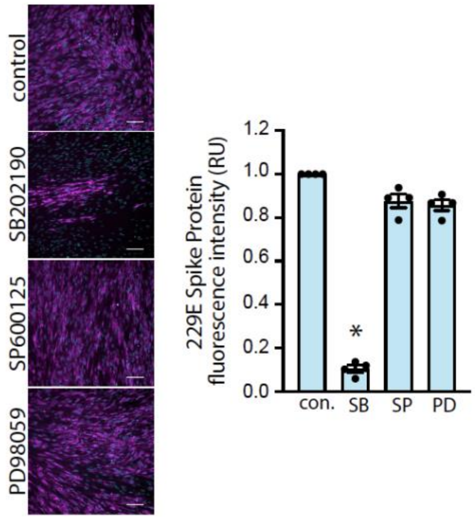

Figure S4. Inhibition of the p38 MAPK recapitulates the effects of capmatinib and IRAK1/4 inhibition on coronavirus infection. (left) Representative images from MRC-5 cells treated with 10 µM SB202190 (SB, p38 MAPK inhibitor), 10 µM SP600125 (SP, JNK inhibitor) or 10 µM PD98059 (PD, MEK1 inhibitor) and infected with 229E for 2 days. (right) Quantification of 229E S protein expression (>10 images per condition, n = 4).
